# Arsenic bioaccumulation in fish of the lower meghna river: Seasonal dynamics, species sensitivity, and public health implications

**DOI:** 10.1371/journal.pone.0330602

**Published:** 2025-09-03

**Authors:** Shyamal Kumar Paul, Nusrat Jahan, Debasish Saha, Bhakta Supratim Sarker, Priyanka Rani Majumdar, Moshiur Rahman

**Affiliations:** 1 Department of Fisheries and Marine Science, Faculty of Biological Science, Noakhali Science and Technology University, Noakhali, Bangladesh; 2 School of Biotechnology and Biomolecular Sciences, UNSW Sydney, Sydney, New South Wales, Australia; 3 International Studies in Aquatic Tropical Ecology (ISATEC), University of Bremen, Bremen, Germany; Bangladesh Council of Scientific and Industrial Research, BANGLADESH

## Abstract

The Lower Meghna River (LMR), located in one of Bangladesh’s most arsenic-contaminated regions, is essential for local fisheries and provides water for drinking, irrigation, and daily use. Consequently, this study investigates arsenic accumulation in ten edible, small indigenous species (SIS) of fish, considering their morphology, habitats, diets, and water and sediment conditions. Samples were analysed across three distinct river segments during three seasons. The arsenic levels in water and sediment fell within non-polluting limits; however, certain fish species, including *Mystus vittatus, Glossogobius giuris, Lepidocephalichthys guntea, Neotropius atherinoides, and Apocryptes bato*, surpassed the WHO’s safe consumption threshold (1 mg/kg). Arsenic levels in water, sediment, and fish show significant seasonal variations (*p <* 0.05) but no notable spatial differences (*p* > 0.05). Strong correlations exist between arsenic in fish and both water and sediment (R^2^ > 0.5). Fish body shape and the presence of scales notably influence arsenic accumulation. Benthic carnivores accumulate more arsenic than pelagic and benthic-pelagic omnivores. Children are especially vulnerable to health risks. While Hazard Indices (HI) and Hazard Quotients (HQ) for water and sediment remain within safe limits, fish consumption presents a risk. Furthermore, the cancer risk associated with fish consumption is higher than that from water and sediment pathways. These results highlight a significant food safety concern and emphasize the need for integrated arsenic risk management strategies in the Lower Meghna River region.

## Introduction

Bangladesh ranks third globally in open-water fisheries production, with Small Indigenous Species (SIS) fish being particularly notable for their high species diversity, strong market demand, and significant nutritional value [1]. Although floodplains, lakes, and rivers all contribute to SIS availability, rivers in particular provide a consistent year-round supply [2]. However, overexploitation and environmental degradation—especially pollution, are contributing to a continual decline in SIS production. Among various pollution sources, industrial effluents and arsenic-contaminated groundwater introduce significant levels of trace metals into river systems [3]. These trace metals interact differently with environmental media and accumulate in aquatic organisms. Fish—especially SIS—are susceptible to trace metal accumulation, which adversely affects their ecological, biological, and physiological functions [4]. Protein source, and fish habitats are at risk due to trace metal pollution [4–8]. This is of particular concern in Bangladesh, where fish serve as the primary protein source and are increasingly at risk due to trace metal pollution [4–8].

The Meghna River, a major waterway in Bangladesh and part of the Ganges Delta, originates from the Barak River in eastern India and enters the country through the Kishoreganj district, supplying a wide variety of small indigenous fish species (SIS) [9]. At Chandpur, the Meghna meets the Padma and later the Jamuna, forming the Lower Meghna, which flows through Laxmipur and Noakhali districts before emptying into the Bay of Bengal [10]. The lower Meghna is almost 153 km long, with 40,532.9 m^3^/s of discharge water [11,12]. The Lower Meghna River supports diverse flora and fauna, including finfish, crustaceans, and shellfish, and holds strategic importance for industrial activities and navigation. However, it is heavily polluted by upstream discharge and local runoff. Notably, the surrounding districts are among Bangladesh’s most arsenic-contaminated groundwater regions, with concentrations reaching 50 μg/L—five times the WHO-recommended limit [13].

Fish accumulate toxic metals from contaminated environments, with accumulation rates varying by species due to differences in metal uptake (via water, sediment, or diet) and elimination mechanisms [4,14]. Additionally, seasonal and spatial variations in anthropogenic pollution levels affect trace metals mobility and speciation [15]. Metals accumulate in fish through several pathways: ion exchange into lipophilic tissues (e.g., gills), ingestion of particulate matter from water, consumption of contaminated food, and adsorption onto skin and tissues [16]. Considering this, fish are noted as one of the indicators for aquatic pollution, as they live in both the water columns and the deep sediment floor [17]. Moreover, their diverse feeding behaviors make them effective bioindicators of environmental pollution [5,6]. Numerous studies have examined the extent to which fish accumulate hazardous trace metals in different organs, with species morphology influencing accumulation based on behavior and habitat exposure. Fish morphology can affect accumulation rates due to differences in movement and water exposure. Studies show that among various exposure routes, dietary intake is the primary way humans are exposed to arsenic, mainly through consuming contaminated fish [4]. Therefore, analyzing arsenic accumulation in small indigenous species (SIS) is essential to understand its transfer to humans through consumption and to develop health risk indices for use in environmental monitoring and policy decisions.

Scientists have long studied arsenic in aquatic environments and organisms due to its potential to cause harmful health effects and expose humans to carcinogens [8,18]. Following this trend, numerous studies have focused on water, sediment, and fish, with the majority conducted in the downstream Meghna River estuary [3]. Moreover, several studies have reported only the enrichment of trace metals in water, sediment, and fish along the river [7,8,19–21]. Recent research has further highlighted the health risks associated with arsenic ingestion through fish consumption and demonstrated significant public health concerns linked to arsenic-contaminated aquatic food sources [22,23]. Moreover, Seasonal and ecological factors, such as trophic behavior, strongly influence arsenic bioaccumulation patterns in freshwater species [24,25]. Additionally, studies have emphasized the ecological risks of arsenic in estuarine systems of Bangladesh and highlight the importance of integrating biological traits with spatial variability in contamination assessments [26–29]. These findings provide a foundation for this study, which aims to evaluate how fish morphometrics and environmental factors jointly affect arsenic uptake. Despite these advances, the roles of seasonality, spatial variation, and trophic position in arsenic accumulation among Small Indigenous Species (SIS) remain poorly understood, though they likely drive bioaccumulation dynamics. Moreover, fish body size and shape may influence arsenic levels in muscle tissue, a hypothesis that warrants detailed investigation. Importantly, no comprehensive human health risk assessment related to arsenic exposure via fish consumption has been conducted for the Lower Meghna River, despite its status as one of Bangladesh’s most arsenic-contaminated groundwater regions. Therefore, this study addresses a critical gap by quantifying arsenic concentrations in commonly consumed SIS and evaluating the associated health risks, providing essential data for risk management and policy development in this high-risk area.

Despite previous studies on arsenic contamination in the Meghna River, most have focused on isolated environmental media or lacking temporal and spatial context. Notably, the combined influence of seasonal fluctuations, spatial variability across multiple sites, and ecological traits on arsenic bioaccumulation in small indigenous fish species (SIS) remains insufficiently unexplored. Additionally, limited research has incorporated fish morphometric and ecological traits into arsenic accumulation assessments. This study addresses these gaps by examining how seasonal, spatial, ecological, and morphometric factors influence arsenic bioaccumulation in SIS. Unlike earlier work focused primarily on chemical concentrations or single-factor assessments, our approach examines how environmental and biological factors jointly influence arsenic uptake. By linking environmental contamination with species-specific traits and human health risk, this research supports more effective monitoring and management strategies in arsenic-impacted river systems. Therefore, the study aimed to (1) determine arsenic concentrations and pollution status in water, sediment, and selected SIS fish muscle tissues (FMTs) in the Lower Meghna River, (2) evaluate how arsenic concentrations vary across seasons and locations, (3) investigate whether fish body shape, habitat, and feeding behavior influence arsenic bioaccumulation in muscle tissue, and (4) assess the probable human health risks from the uptake of arsenic through different pathways. This research will offer a comprehensive pre-assessment to support policymakers in environmental and health-related decision-making. To support clarity and accessibility for a broader audience, a glossary of key technical terms and abbreviations is included as Supplementary Information (S1 Glossary).

## Materials and methods

### Selection of sampling sites, sample size

**Fig 1:**
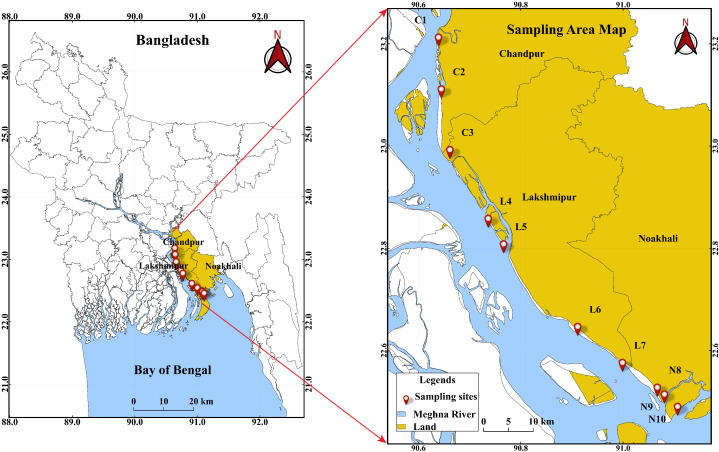
Locations for sampling along the Lower Meghna River in Bangladesh’s Chandpur, Lakshmipur, and Noakhali District. The map was created using QGIS (version 3.42.0) and shapefiles obtained from the Humanitarian Data Exchange (HDX): Common Operational Datasets – Administrative Boundaries for Bangladesh (https://data.humdata.org/dataset/cod-ab-bgd). This figure is original and published under a CC BY 4.0 license.

Ten sampling zones were established across the three districts, covering nearly all major fishing areas (Fig 1). Chandpur was designated as the upper zone (the confluence of three rivers), Lakshmipur as the mid zone, and Noakhali as the lower zone due to its proximity to the estuary. Accordingly, sampling points C1–C3 represent the upper zone, L4–L7 the mid zone, and N8–N10 the lower zone. To assess seasonal variations, samples were collected during the pre-monsoon (March), monsoon (June), and post-monsoon (October) periods. The study was conducted from July 2022 to June 2023. Fish specimens were obtained from local fishermen using cast nets. Water samples were collected in pre-cleaned dark polyethylene bottles from approximately 50 cm below the surface, while sediment samples were collected using a hand-driven corer (0–10 cm depth) to retrieve the surface layer [18,30]. To minimize sampling bias, three replicate samples of water and sediment were collected at each station, maintaining a minimum distance of 1 km between sampling points. The replicates were pooled to produce a single composite sample per station for each season. For fish sampling, ten individuals—one per species—were obtained for each of the ten selected small indigenous species (SIS) at every station during each season. This approach resulted in a total of 30 water samples, 30 sediment samples, and 300 fish samples (10 species × 10 stations × 1 fish per species × 3 seasons). The same ten SIS species were targeted across all stations; if a species was unavailable at a particular location, specimens were collected from the nearest adjacent station to ensure consistency. Detailed information on the selected SIS fish, including sample IDs, morphometric traits (body shape, scale presence, and maximum length), feeding behavior, and habitat types, is provided in S1 Table.

### Water and sediment samples collection and preparation

Surface water samples were collected manually at a depth of 0.5 m using 0.5 L dark polyethylene bottles. The samples were immediately transported to the laboratory and filtered through a Millipore assembly equipped with a 0.45 μm membrane filter [31]. After filtration, samples were acidified with 2 mL of HNO3 per litre and stored at 4°C until analysis [14]. Arsenic concentrations in water were determined by inductively coupled plasma mass spectrometry (ICP-MS, Elan 9000, PerkinElmer, Germany). The samples were calibrated with a standard multi-element solution (Merck, Germany).

Surface sediment samples (0–10 cm depth) were collected manually using a mud corer over a 1 × 1 m area. To minimize contamination, the top 2 cm of each sample was removed with a sterile plastic spatula [32]. Samples were placed in pre-cleaned plastic bags (10% nitric acid) and stored at 4°C until further processing [15]. In the laboratory, sediments were dried at 50°C until constant weight, then pulverized using a mortar and pestle and homogenized in an agate mortar. The processed samples were stored in glass bottles [32–34]. Samples were ignited in a muffle furnace, with temperature increased gradually to 450°C at 50°C/h to avoid organic loss. After 8 hours, the samples were digested with 50% nitric acid. The digested samples were subsequently filtered through Whatman No. 44 filter paper (mesh size 20 µm) to separate fine sediment particles before chemical analysis in a 50 mL chemical flas [15]. Arsenic concentrations in sediments were determined using atomic absorption spectroscopy (SHIMADZU AA-7000) following standard analytical procedures [32,35].

Analytical blanks and certified reference materials (CRM 320, Merck KGaA, Germany) were used to validate accuracy and precision. Recovery rates for arsenic ranged from 95% to 105%, with relative standard deviation (RSD) below 10%, indicating high data reliability [30]. For enhanced accuracy, all plastic bottles were pre-washed with 10% HNO3 for at least 48 hours, and only reagents of analytical grade or higher were used [15]. The detection limits for arsenic were 0.001 mg/L in water and 0.001 mg/kg in sediments [14].

### Fish sample collection and preparation

After collecting fish samples, they were washed thoroughly with tap water to remove soil and other particles. Subsequently, the samples were rinsed with de-ionized water. The samples were then stored at −20°C until further analysis. Fish muscle tissue (FMT) samples were minced and mixed well prior to arsenic testing. After dissection, 2 g of muscle tissue from each sample was taken for subsequent analysis. The samples were then freeze-dried for 24 hours, and 0.5 g aliquots were taken from each dried fraction. In this instance, the Zirbus freeze-drying equipment (Model: VaCo 2, Germany; free of CFC; condenser dimensions: Ø200 × 200 mm; volume: 5.7 L; ice capacity: 2 kg/24 h; cooling system: single stage) was utilized [36]. Following drying, the aliquots were analyzed using ICP-MS (Model: ELAN9000, PerkinElmer, Germany) to determine metal concentrations. Additionally, 0.5 g of muscle sample was digested using a Microwave Digestion System (WX-6000, China) with 10 mL of ultrapure HNO_3_. The digested solution was filtered and then diluted in a 50 mL Teflon (tezaron) tube for two hours prior to analysis [3]. The protocol included standard reference materials and analytical blanks. Arsenic concentration was determined using a standard solution (Mark VI, Germany) prepared in the same acid matrix [37]. To ensure the precision, accuracy, and validity of the analytical method, certified reference material (CRM 320, Merck KGaA, Germany; N = 3) was used [3].

### Ethical statement

Samples from wild populations were obtained through local fishermen. All specimens collected were of commercial size and marketable condition, consistent with those typically consumed by the local population. All sampled species were neither endangered nor protected. Consequently, no permits were required for the execution of this study. Furthermore, there were no ethical concerns associated with the experimental procedures as there were no harmful or invasive methods used. All sampling was conducted responsibly ensuring minimal disturbance to the environment and animal welfare. Moreover, no live animals were handled or sacrificed by the researchers, and no procedures involving anesthesia, analgesia, or euthanasia were conducted. As such, no experimental manipulation or interventions were performed.

### Inclusivity in global research

Additional information regarding the ethical, cultural, and scientific considerations specific to inclusivity in global research is included in the Supporting Information (S1 Checklist).

### Quality assurance and quality control

Stringent QA/QC protocols were applied to ensure accurate arsenic determination in fish, water, and sediment samples. All labware was acid-cleaned to prevent contamination, and samples were analyzed in batches with method blanks. Calibration curves from multi-level arsenic standards exhibited excellent linearity (R² > 0.998). NIST SRM 2702 for sediment and NIST SRM 2976 for fish tissue and arsenic standard solutions for water, were used to validate analytical accuracy, with recoveries between 93% and 106%. Duplicate analyses (10% of samples) demonstrated high precision with relative standard deviations ≤ 11%. Limits of detection (LOD) and quantification (LOQ) were established for each matrix based on replicate blanks: fish muscle tissue LOD = 0.02 μg/g, LOQ = 0.06 μg/g wet weight; sediment LOD = 0.5 μg/kg, LOQ = 1.5 μg/kg dry weight; and water LOD = 0.1 μg/L, LOQ = 0.3 μg/L. These detection limits were adequate for measuring environmental arsenic concentrations. Regular instrument calibration and maintenance ensured consistent performance throughout the study.

### Potential environmental and ecological risk

#### Arsenic pollution indices for water media.

Several standard indices were used to assess water pollution levels at each sampling site, including the Heavy Metal Pollution Index (HPI) [38,39], Contamination Degree (CD) [40], and Nemerow Pollution Index (NPI) [41]. These indices help quantify the extent of arsenic contamination relative to environmental safety thresholds. Their classification criteria are provided in S2 Table.


$$HPI=\frac{\sum_{i=1}^{n}\left(\frac{{MC}_{i}}{{SC}_{i}}\times 100\right)\times \frac{1}{{SC}_{i}}}{\sum_{i=1}^{n}\frac{1}{{SC}_{i}}}$$ (1)

$$CD=\sum_{i=1}^{n}\frac{{MC}_{i}}{{SC}_{i}}$$ (2)

$$NPI=\sqrt{\frac{{{\left(\frac{{MC}_{i}}{{SC}_{i}}\right)}_{mean}^{2}+\;\left(\frac{{MC}_{i}}{{SC}_{i}}\right)}_{maximum}^{2}}{2}}$$ (3)

Here, MC_i_ and SC_i_ represent the measured and standard concentrations of arsenic in river water (S2 Table); n is the number of target metals, which is 1 in our study. The SC_i_ value in this study was taken from the Environmental Conservation Rules (DoE), Bangladesh (Table 1) [42], which sets the maximum standard concentration of arsenic in freshwater at 0.05 mg/l. For NPI, we considered the mean and Maximum values from three seasons in each sampling site.

**Table 1:** Seasonal and spatial variation of trace metal concentrations in the water (mg/l), sediment (mg/kg), and 10 SIS fish (mg/kg) of Lower Meghna River, Bangladesh. One-way ANOVA indicates the significant difference between samples through Seasonal and Spatial variation.

Sample	Statistics	Seasonal variation				Spatial variation			
		Pre-Monsoon (n = 10)	Monsoon (n = 10)	Post-Monsoon (n = 10)	p-Value	Upper-part (n = 9)	Mid-part (n = 12)	Lower-part (n = 9)	p-Value
Water	Range	0.0084-0.02	0.0009-0.0031	0.0025-0.0082	0.0	0.002-0.018	0.001-0.015	0.001-0.013	0.89
	Mean±SD	0.012 ± 0.003^a^	0.002 ± 0.0007^c^	0.006 ± 0.0018^b^		0.007 ± 0.005	0.006 ± 0.004	0.006 ± 0.004	
	CV (%)	25.76	38.96	32.57		73.950	72.340	60.846	
Sediment	Range	5.49-8.43	4.05-5.91	4.73-6.70	0.0	4.99-7.16	4.77-8.43	4.05-8.32	0.5
	Mean±SD	6.88 ± 0.98^a^	4.94 ± 60^b^	5.55 ± 70^b^		6.03 ± 0.72	5.88 ± 1.09	5.43 ± 1.29	
	CV (%)	14.24	12.17	12.61		11.97	18.59	23.84	
Fish									
Mv	Range	0.53-1.03	0.23-0.58	0.31-0.76	0.0	0.33-1.03	0.30-0.89	0.23-0.96	0.19
	Mean±SD	0.82 ± 0.16^a^	0.35 ± 0.10^c^	0.52 ± 0.13^a^		0.69 ± 0.22	0.53 ± 0.20	0.48 ± 0.23	
	CV (%)	19.78	30.10	24.78		32.73	38.08	46.91	
Ps	Range	0.28-0.54	0.12-0.28	0.20-0.44	0.0	0.12-0.54	0.12-0.40	0.17-0.52	0.25
	Mean±SD	0.40 ± 0.09^a^	0.20 ± 0.05^c^	0.29 ± 0.07^b^		0.34 ± 0.13	0.26 ± 0.07	0.29 ± 0.10	
	CV (%)	23.20	25.94	23.28		37.01	28.33	35.27	
Gg	Range	0.76-1.15	0.36-0.55	0.65-0.87	0.0	0.36-1.06	0.39-1.15	0.42-1.06	0.97
	Mean±SD	0.92 ± 0.13^a^	0.45 ± 0.06^c^	0.76 ± 0.08^b^		0.72 ± 0.23	0.69 ± 0.22	0.72 ± 0.20	
	CV (%)	14.62	13.52	10.61		31.78	31.67	27.50	
Am	Range	0.40-76	0.20-0.42	0.33-0.55	0.0	0.26-0.76	0.25-0.69	0.20-0.70	0.43
	Mean±SD	0.59 ± 11^a^	0.28 ± 0.07^c^	0.44 ± 0.07^b^		0.48 ± 0.15	0.43 ± 0.12	0.39 ± 0.17	
	CV (%)	19.35	25.69	15.39		31.76	28.21	42.63	
Dd	Range	0.53-0.87	0.29-0.65	0.47-0.77	0.0	0.39-0.87	0.38-0.85	0.29-0.75	0.16
	Mean±SD	0.70 ± 0.12^a^	0.44 ± 10^b^	0.60 ± 11^a^		0.63 ± 0.14	0.60 ± 0.14	0.50 ± 0.13	
	CV (%)	17.47	23.52	18.17		22.12	23.91	26.63	
Lg	Range	0.79-1.17	0.37-0.73	0.60-0.85	0.0	0.54-1.10	0.49-1.17	0.37-1.05	0.39
	Mean±SD	0.95 ± 0.13^a^	0.54 ± 11^c^	0.74 ± 0.09^b^		0.80 ± 0.16	0.75 ± 0.19	0.67 ± 0.22	
	CV (%)	13.82	20.40	11.49		19.75	25.32	32.34	
Cc	Range	0.49-0.78	0.17-0.32	0.29-0.58	0.0	0.19-0.74	0.17-0.78	0.21-0.62	0.98
	Mean±SD	0.61 ± 0.09^a^	0.24 ± 0.05^c^	0.42 ± 0.08^b^		0.43 ± 0.18	0.43 ± 0.17	0.41 ± 0.15	
	CV (%)	15.53	19.28	19.63		42.47	40.78	35.50	
Gc	Range	0.50-0.87	0.22-0.44	0.29-0.67	0.0	0.36-0.87	0.27-0.77	0.22-0.64	0.18
	Mean±SD	0.66 ± 0.11^a^	0.31 ± 0.07^c^	0.44 ± 0.12^b^		0.55 ± 0.16	0.47 ± 0.18	0.40 ± 0.13	
	CV (%)	16.33	21.19	27.23		28.13	39.14	33.66	
Na	Range	0.80-1.17	0.28-0.54	0.47-0.85	0.0	0.38-1.17	0.28-1.16	0.30-1.11	0.84
	Mean±SD	0.97 ± 0.15^a^	0.38 ± 0.09^c^	0.65 ± 0.12^b^		0.71 ± 0.24	0.65 ± 0.28	0.64 ± 0.26	
	CV (%)	15.38	23.11	18.29		34.44	43.65	40.81	
Ab	Range	0.99-1.67	0.44-0.66	0.77-1.08	0.0	0.44-1.67	0.44-1.47	0.48-1.48	0.86
	Mean±SD	1.35 ± 0.20^a^	0.53 ± 0.08^c^	0.90 ± 0.09^b^		0.98 ± 0.41	0.89 ± 0.34	0.92 ± 0.33	
	CV (%)	15.06	15.13	9.59		41.67	37.97	35.86	

Mv, *Mystus vittatus;* Ps*, Puntius sophore;* Gg*, Glossogobius giuris;* Am*, Amblypharyngodon mola;* Dd*, Devario devario;* Lg*, Lepidocephalichthys guntea;* Cc*, Chela cachius;* Gc*, Gudusia chapra;* Na*, Neotropius atherinoides;* Ab*, Apocryptes bato.*

#### Arsenic pollution indices for Sediment media.

The Geo-accumulation index (I_geo_) [43], the Contamination factor (CF) [44,45], and the Ecological risk index (ERI) [46] were adopted to calculate the pollution level of each sampling site in the sediment media. The following equations were used to calculate the metal pollution indices, and their classification criteria are provided in S2 Table.


$$Igeo={\text{log}}_{2}\left[\frac{{MC}_{i}}{{1.5SC}_{i}}\right]$$ (4)

$$\begin{array}{c} CF=\sum {\mathrm{\backslash nolimits}}_{i=1}^{n}\frac{{MC}_{i}}{{SC}_{i}} \end{array}$$ (5)

$$\begin{array}{c} ERI=\sum {\mathrm{\backslash nolimits}}_{i=1}^{n}Ti\times \frac{{MC}_{i}}{{SC}_{i}} \end{array}$$ (6)

Here, MC_i_ and SC_i_ are the measured and standard concentrations of arsenic in river Sediment (ST2). The SC_i_ value in this study was derived from the average arsenic value in shale sediments (Table 2), which establishes the maximum standard concentration of arsenic in river sediments at 13 mg/kg. For Igeo, factor 1.5 denotes the “background matrix correlation value” responsible for the lithospheric effect in aquatic sediments [32]. In ERI, Ti is the toxicity factor, which is 10 for arsenic [45].

**Table 2:** Comparison of trace metal concentrations in the water (mg/L), sediment (mg/kg), and fish (mg/kg) of Lower Meghna River, Bangladesh, to other studies and standard values.

Country	Sampling site	Water (mg/l)	Sediment (mg/kg)	Fish (mg/kg)	References
Bangladesh	Lower Meghna River	0.001-0.018	4.05-8.43	0.12-1.68	This study
	Upper Meghna River	NA	NA	0.14-0.89	[78]
	Meghna River Estuary	NA	NA	0.75-1.48	[3]
	Meghna River Estuary	NA	NA	0.760	[79]
	Meghna River Estuary	0.012–0.039	NA	NA	[30]
	Kirtankhola River	0.002-0.007	2.09-6.89	0.05-1.38	[6]
	Karnaphuli River	NA	NA	0.86-5.39	[5]
	Gumti River	NA	0.01-0.04	NA	[32]
	Paira River	NA	NA	0.04-1.06	[80]
	Turag River	0.007-0.02	NA	0.09-0.28	[33]
	Buriganga River	0.01-0.02	NA	0.09-0.36	[33]
	Shitalakha River	0.008-0.02	NA	0.12-0.22	[33]
	Dhaleshwari River	0.21-1.02	4.20-7.25	< 0.002	[65]
	Bhairab River	0.002-0.005	2.96-5.32	NA	[66]
	Halda river	NA	4.36-9.40	NA	[67]
India	Kalpakkam cost	< 0.002	< 0.002	< 0.002	[81]
	Thamirabarani River	NA	0.94-2.06	0.002-0.27	[82]
China	Xinfengjiang River	NA	3.45-437	NA	[68]
	Dianchi Lake	0.02-0.11	20.0-41.5	0.29-0.47	[77]
Iran	Miankaleh Peninsula	0.005-0.027	1.5-6.9	4.0-5.8	[83]
	Chabahar Bay	NA	3.2–9.7	0.47	[84]
Nigeria	Niger River	< 0.002- 0.005	< 0.002	0.002-0.09	[85]
Ghana	Densu River	< 0.002- 0.12	0.09-1.875	0.02-0.18	[69]
Lesotho	Caledon River	NA	1.87-2.81	1.08-1.5	[86]
Ethiopia	Lake Hawassa	0.0001-0.002	1.2-68.3	0.06–1.82	[87]
Guidelines					
CCC (Criterion continuous concentration)		0.15			[57]
CMC (Criteria maximum concentration)		0.34			
US-EPA (Drinks)		0.01			[88]
US-EPA (Aquatic Life)		0.15			
WHO		0.01		1	[73]
Environmental Conservation Rules (DoE), Bangladesh		0.05			[42]
Average shale Value			13		[89]
TRV (toxicity reference value)			6		[66]
LEL (lowest effect level)			6		[90]
SEL (severe effect level)			33		
US-EPA				1.3	[72]
Ministry of Fisheries and Livestock, Bangladesh				5	[74]

#### Metal Bioaccumulation factor (MBAF).

Bioaccumulation Factor denotes the ability of an organism to accumulate metals from its surrounding environment [6]. It reflects the amplitude of the metal concentration levels in an organism’s accumulation organs throughout time [3]. Water and sediment media considered for analysing MBAF through the following equation proposed by the researchers for Fish [47–49]:


$${MBAF}_{water}=\frac{C_{Fish}}{C_{water}}$$ (7)

$${MBAF}_{sediment}=\frac{C_{Fish}}{C_{sediment}}$$ (8)

C_Fish,_ C_Water, and_ C_sediment_ represent the metal concentrations in FMT (mg/kg), the water medium (mg/L), and the sediment medium (mg/kg), respectively. Arnot & Gobas categorized MBAF_water_ values into the following ranges: MBAF_water_ < 1000: less probability of accumulation; 1000 < MBAF_water_ < 5000: bioaccumulative; MBAF_water_ > 5000: highly bioaccumulative [50]. MBAF_sediment_>1 indicates bioaccumulative from the sediment, wherein the organism’s metabolism/excretion of the contaminant is ineffective [51].

### Human health risk assessment

#### Estimated daily intake (EDI).

According to the US Environmental Protection Agency (US EPA), using health risk assessment is a suitable method to ascertain the harmful effects of exposure to trace elements on health [52]. So, the human health risk depends on the foodstuffs taken, the water they drink, the air they inhale daily, and the level of the metals in these media [6,14,32]. Formulas from the US EPA were used to assess health risks from Inhalation, Ingestion, and Dermal contact of arsenic [53–55].


$$\text{EDIw }(\text{ingestion})=\;\frac{\text{CN }\times \text{ EF }\times \text{ ED }\times \text{ IRw}}{\text{BWt }\times \text{ AT}\times \;10^{6}}$$ (9)

$$\text{EDIw }(\text{Dermal})=\;\frac{\text{CN }\times \text{ SA }\times \text{ KC }\times \text{ EF }\times \text{ ED }\times \text{ ET }\times \text{ ABS}}{\text{BWt }\times \text{ AT }\times \;10^{6}}$$ (10)

$$\text{EDIs }(\text{ingestion})=\;\frac{\text{CN }\times \text{ EF }\times \text{ ED }\times \text{ IRS}}{\text{BWt }\times \text{ AT}\times \;10^{6}}$$ (11)

$$\text{EDIs }(Dermal)=\;\frac{\text{CN }\times \text{ SA }\times \text{ AF}\times \text{ EF }\times \text{ ED }\times \text{ ABS}}{\text{BWt }\times \text{ AT }\times \;10^{6}}$$ (12)

$$\text{EDIs }(\text{Inhalation})=\;\frac{\text{CN }\times \text{ PM }\times \text{ ET }\times \text{ EF }\times \text{ ED }\times \text{ IRair}}{\text{BWt }\times \text{ PEF }\times \text{ AT}}$$ (13)

$$\text{EDIf }(\text{Ingestion})=\;\frac{\text{CN }\times \text{ IR}f}{\text{BWt}}$$ (14)

The explanations and values for the estimated daily intake (EDI) pathways used are listed in S3 Table.

#### Non-carcinogenic risk assessment.

Owing to varying exposures to an individual’s trace metal contents, the hazard quotient (HQ) for a given element was evaluated to calculate the risk index (HI), which is the non-carcinogenic risk [32]. The hazard quotient (HQ) quantifies the degree of non-carcinogenic health risk exposure to toxic metal components in particular media: Water, sediment and Fish [3,30,32]. On the other hand, the overall health risk (HI) is calculated by summing the HQ values across all applicable pathways [14].


$${HQ}_{ip}=\frac{{EDI}_{ip}}{{RfD}_{ip}}$$ (15)

$$HI=\;\sum_{i=l}^{n}{HQ}_{ip}=\;HQinhalation+HQdermal+HQingestion$$ (16)

Where EDIip represents how much arsenic a person consumes daily through water, sediment, or fish; RfDip is the reference dose of arsenic for distinct pathways of adults and children [32], provided by the USEPA for Dermal; 0.0003 [56,57]. When the measured value exceeds 1, the receptors will have a non-carcinogenic health effect [30]. When the HI value exceeds 1, individuals may be exposed to non-carcinogenic consequences, which are expected to worsen in the absence of effective remediation strategies [32].

#### Carcinogenic risk assessment.

To determine whether a specific carcinogen may cause cancer in an individual, a carcinogenic risk assessment needs to be carried out [30]. According to the available specific slope factor (CSF) of carcinogens, the results discuss the risk of exposure [58].


$${CR}_{ip}={CSF}_{ip}\times {EDI}_{ip}\;$$ (17)

$$CR=\;\sum_{ip=l}^{n}{CR}_{ip}$$ (18)

Where CRip represents the carcinogenic risk for arsenic through its specific pathway, while CSFip refers to the slope factor for arsenic, which determines the probability of cancer development [59]. CSFip is 1.5 mg/ kg/day for arsenic [60,61]. The acceptable range for lifetime exposure to carcinogenic risk (CR) typically falls between 10 ⁻ ⁶ and 10 ⁻ ⁴ [62,63]. A CR value exceeding this limit suggests an increased probability of potential carcinogenic risk [56,57,64].

### Statistical analysis

All statistical analyses were conducted using R version 4.2.2 (R Core Team, 2022). Data visualization and statistical interpretation were carried out with the help of packages including *ggplot2, dplyr, stats, FactoMineR*, and *ggpubr.* Prior to conducting parametric tests, the Shapiro–Wilk and Kolmogorov–Smirnov tests were applied to assess normality. When the assumptions of normality and homogeneity of variance (checked using Levene’s test from the car package) were not met, non-parametric alternatives such as the Kruskal–Wallis test were used instead of ANOVA. One-way ANOVA was used to assess differences in arsenic concentrations among seasons, sampling sites, and fish species, followed by Tukey’s HSD post hoc test to determine pairwise differences. For morphometric factors (e.g., body shape, trophic position), independent t-tests and linear regression analyses were applied, where applicable. The significance level was set at p ≤ 0.01 or 0.05 depending on the test context. Principal Component Analysis (PCA) was used to identify patterns and spatial-seasonal grouping based on arsenic concentrations in water, sediment, and fish muscle tissue (FMT). A correlation matrix was computed to assess co-association between arsenic levels across compartments. All models were visually inspected using residual plots to validate assumptions such as linearity and homoscedasticity where relevant. Figures presenting boxplots, bar graphs, and PCA biplots were created using *ggplot2* and enhanced with *ggfortify* and *factoextra*. This approach ensured robust analysis while addressing variability in the data, enhancing reproducibility and statistical rigor in our findings.

## Result and discussion

### Spatiotemporal distribution of arsenic in water

**Fig 2:**
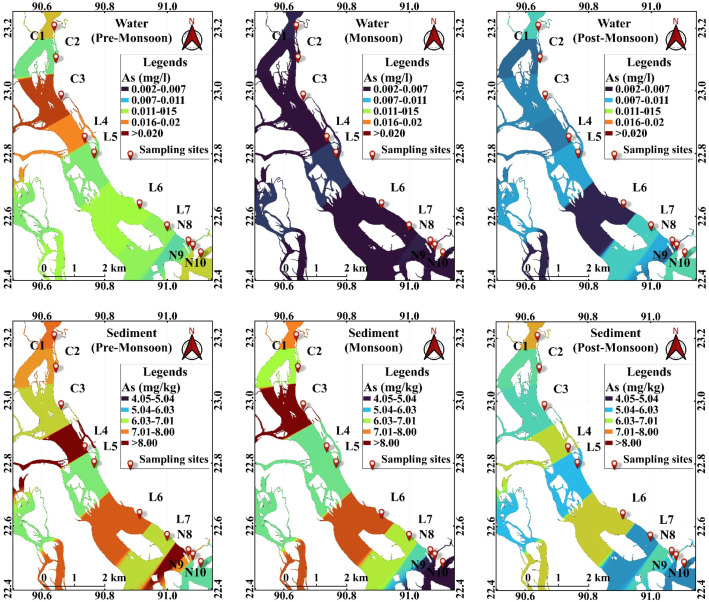
Spatial distribution and concentration of Arsenic in water and sediment along the studied Lower Megna River. The map was created using QGIS (version 3.42.0) and shapefiles obtained from the Humanitarian Data Exchange (HDX): Common Operational Datasets – Administrative Boundaries for Bangladesh (https://data.humdata.org/dataset/cod-ab-bgd). This figure is original and published under a CC BY 4.0 license.

The arsenic concentration in Water is demonstrated in Table 1. The mean ± SD arsenic concentration varied significantly across seasons (p < 0.01), indicating strong seasonal influence on arsenic mobility in aquatic systems. Arsenic concentration was highest in the pre-monsoon season (0.012 ± 0.003 mg/L), followed by post-monsoon (0.006 ± 0.0018 mg/L) and monsoon (0.002 ± 0.0007 mg/L) periods. Spatial differences in arsenic concentration were not statistically significant (p > 0.01) (Table 1). Despite statistical insignificance, a decreasing trend was observed from Upper-part (0.007 ± 0.005 mg/L) to Mid-part and Lower-part (both 0.006 ± 0.004 mg/L) (Fig 2). The minimum arsenic concentration in pre-monsoon was at N8 (Lower-part), whereas the maximum concentration was at C3 (Upper-part). Unlike pre-monsoon, maximum and minimum arsenic concentrations were found in the mid-part at L5 and L6, respectively. Due to the Monsoon, the minimum concentration (0.0025 mg/L) of arsenic was found in L6; however, the maximum concentration (0.0082 mg/L) was observed at N10 during the post-monsoon period. The spatial distribution of arsenic may be influenced by both anthropogenic discharges and natural geogenic sources, particularly during low-flow periods. This seasonal pattern aligns with recent reports that arsenic concentration in water increases during the dry season due to lower dilution and higher evaporation [24,30]. Bangladesh’s pre-monsoon season experiences less precipitation, resulting in a higher concentration of arsenic during that time. Compared to other rivers nationally and internationally, arsenic concentrations in this study were either similar or lower, except for heavily polluted sites such as the Dhaleshwari River (Table 2). Moreover, the arsenic concentration was under the recommended values for drinking aquatic life survival set by the US-EPA, WHO and DoE (Bangladesh).

### Spatiotemporal distribution of arsenic in sediment

The mean ± SD arsenic concentration significantly differed in pre-monsoon sediments compared to other seasons (*p <* 0.01) (Table 1). Specifically, the sediment pool accumulated a higher concentration of arsenic in the pre-monsoon period (6.88 ± 0.98 mg/kg) than in the monsoon (4.94 ± 0.60 mg/kg) and post-monsoon periods (5.55 ± 0.70 mg/kg). Like water, sediments exhibited no significant difference refers to either the seasons or space (*p >* 0.01) (Table 1). Moreover, the nearer the Estuary, the lower the arsenic concentration in sediment: Upper-part (6.03 ± 0.72 mg/kg)>Mid-part (5.88 ± 1.09 mg/kg)>Lower-part (5.43 ± 1.29 mg/kg) (Fig 2). Interestingly, the minimum concentration of arsenic in sediment was found at N10 throughout the year. For maximum concentrations, the Monsoon and Post-Monsoon periods were at the upper levels (C3 and C1, respectively). Moreover, the highest concentration site in Pre-Monsoon was somewhat lower than the other two seasons at the Mid-Part (L4). Our results were consistent with those of previous studies, as shown in Table 2 [6,32,65–67]. However, all the rivers studied, except Halda (4.36–9.40 mg/kg) in Bangladesh, had arsenic concentrations in sediment below those of this study. On the other hand, the highly polluted Xinfengjiang River [68] and Dianchi Lake [69] in China, as well as Lake Hawassa (Melake et al., 2022) in Ethiopia, had concentrations 3–50 times higher than those in our study (Table 2). Arsenic values in riverine bed sediments varied from 1 to 55.2 mg/kg, whereas less than 10 mg/kg is considered to be from natural sources [70]. Our findings indicate that the arsenic in the sediments mainly came from natural sources. The seasonal variation for arsenic in sediment supports the finding in Taihu Lake, China, where the highest concentrations were in the pre-monsoon [71]. The authors explained that the cause, as the Pre-Monsoon season has the lowest water flux to carry the new sediment and a higher settling rate of arsenic from water to sediment, has demonstrated a higher concentration. Spatially, the insignificant variation of arsenic may be due to the small sampling area or the absence of significant anthropogenic sources. However, Although the arsenic concentration in sediments was below the average shale value (13 mg/kg), some sampling sites exceeded the TRV (toxicity reference value) and LEL (lowest effect level) value (6 mg/kg), indicating prospecting toxically situation for the organisms living in the sediments [32].

### Spatiotemporal distribution of arsenic in FMT

**Fig 3:**
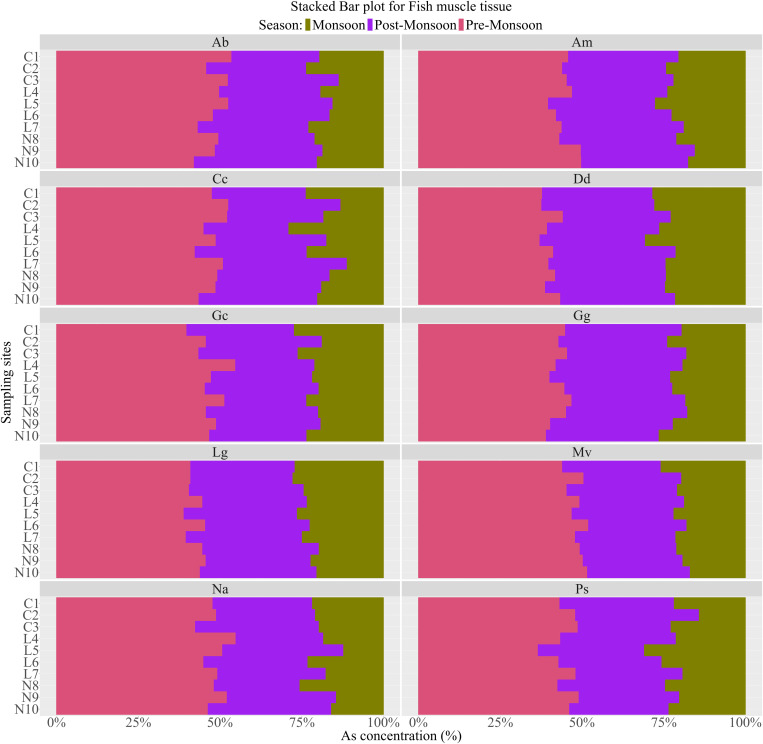
Stacked Bar plot for Arsenic concentration in Ten SIS FMT at three sampling sites: Upper-part (C1-C4), Mid-part (L4-L7) and (N8-N10) for three seasons. Arsenic concentrations are expressed as percentages, and seasons are expressed in three colours. Mv, *Mystus vittatus*; Ps, *Puntius sophore*; Gg*, Glossogobius giuris*; Am, *Amblypharyngodon mola*; Dd, *Devario devario*; Lg, *Lepidocephalichthys guntea*; Cc, *Chela cachius*; Gc, *Gudusia chapra*; Na, *Neotropius atherinoides*; Ab, *Apocryptes bato*.

Fish muscle arsenic concentrations also varied significantly by season (p < 0.01) (Table 1), with the highest values detected during Pre-monsoon than in the Monsoon and Post-Monsoon Periods. The decreasing trends of Mean±SD arsenic in Fishes followed as Ab (0.93 ± 0.36 mg/kg)> Lg (0.74 ± 0.20 mg/kg)> Gg (0.71 ± 0.22 mg/kg)> Na (0.67 ± 0.27 mg/kg)> Dd (0.58 ± 0.15 mg/kg)> Mv (0.56 ± 0.23 mg/kg)> Gc (0.47 ± 0.17 mg/kg)> Am (0.43 ± 0.15 mg/kg)> Cc (0.42 ± 0.17 mg/kg)> Ps (0.30 ± 0.11 mg/kg). Spatial variations in fish did not significantly affect arsenic accumulation in FMT (*p >* 0.01) (Table 1). Nonetheless, the fish from the upper part of the river had higher concentrations of arsenic in the lower part and followed the trend as Upper-part (0.64 ± 0.28 mg/kg)> Mid-part (0.57 ± 0.27 mg/kg)> Lower-part (0.54 ± 0.27 mg/kg) (Table 1). Spatially, all the fish demonstrated higher concentrations at the Upper Part to Mid-part: C1 (Ps, Dd, Na), C2 (Am, Gc), C3 (Mv, Ab), L4 (Lg), and L7 (Gg, Cc) in the Pre-Monsoon Period (Fig 3). Meanwhile, they delineated lower concentrations in the Monsoon at the mid-Part to Lower-Part, except for the fish Gg (C1). In the mid-part, the Ab and Na were at L5, and Ps and Cc were at L7, with lower arsenic concentrations. The N8 (Mv, Am, Gc) and N10 (Dd, Lg) were identified for the lower arsenic concentration in the Lower-part.

Several authors argue that changes in fish movement due to seasonal fluctuations significantly impact trace metal accumulation in body tissue [5,33]. This comprehensive research, which considers the seasonal variations and spatial distribution of accumulation in fish tissue, reveals that fish move more during the monsoon season and gradually decrease from post-monsoon to pre-monsoon, leading to a corresponding seasonal variation in arsenic concentration. The spatial variation in accumulation in fish tissue, from upper to lower parts, is possibly due to the settling of Upper-part sediment, which lowers the possibility of accumulation in the fish tissue. The arsenic concentrations in fish observed in this study (0.12–1.68 mg/kg, Table 2) were in line with those of other studies in different rivers across Bangladesh, except for the Karnaphuli River (0.86–5.39 mg/kg). This river receives many untreated effluents from various industries, including the oil refinery, fertiliser, and shipbreaking industries, which are notable sources of arsenic [5]. In contrast, our study area, while influenced by similar industries, exhibited arsenic concentration comparable to other rivers, excluding the heavily polluted Karnaphuli (Table 2). For the fish samples, 7% exceeded the World Health Organization (WHO) guideline for permissible arsenic concentrations (1 mg/kg), while 2% exceeded the U.S. Environmental Protection Agency (US-EPA) guideline (1.3 mg/kg) [72,73]. *Apocryptes bato* was the only species to exceed both WHO and US-EPA limits for arsenic in edible fish tissue. The other fish that exceeded the WHO limit were *Mystus vittatus, Glossogobius giuris, Lepidocephalichthys guntea,* and *Neotropius atherinoides.* However, all the fish were within safe limits according to the guidelines (5 mg/kg) by the Ministry of Fisheries and Livestock, Bangladesh [74].

### Environmental and Morphometric Impact in arsenic accumulation for fish

#### Relationship among water, sediments and FMT.

**Fig 4:**
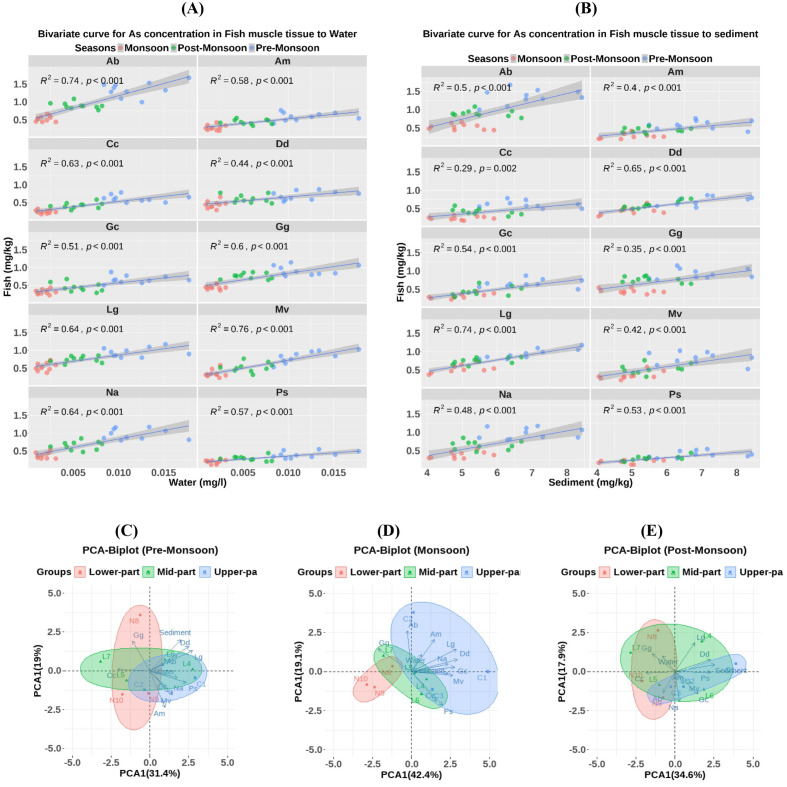
Bivariate relationships of Arsenic concentration between Water versus Fish Muscle tissue (FMT) (A) and sediment versus FMT (B). Principal Component Analysis (PCA) of Arsenic concentration in Fish water and Sediment in Pre-monsoon (C), Monsoon (D), and Post-Monsoon (E). Different colours of dots delineate the season (A and B) and the Sampling Zones **(C-E)**. The p-value indicates the significance level at 0.001. Mv, *Mystus vittatus*; Ps, *Puntius sophore*; Gg, *Glossogobius giuris*; Am, *Amblypharyngodon mola*; Dd, *Devario devario*; Lg, *Lepidocephalichthys guntea*; Cc, *Chela cachius*; Gc, *Gudusia chapra*; Na, *Neotropius atherinoides*; Ab, *Apocryptes bato*.

Fig 4, delineates the relationship between arsenic concentration in water, sediments, and FMT. The bivariate linear regression plot demonstrates that increased arsenic concentration in water significantly increases the arsenic accumulation in FMT (R^2^ > 0.50, p < 0.001) except the fish Dd (R^2^ < 0.50). Among the Fishes, the Mv and Gc demonstrated the highest (R² = 0.76) and lowest (R² = 0.51) significant relationships with water arsenic concentration, respectively. On the other hand, only four fish (Gc, Lg, Dd, and Ps) exhibited a significant relationship with sediment arsenic concentration (R² > 0.50, *p <* 0.001). The seasonal variation in arsenic input into water and sediment may affect the accumulation rate of Fish. Moreover, the habitat zone, feeding habits, and morphological features also significantly varied the accumulated arsenic in FMT. To estimate the impact of seasonal variation on water and sediment accumulation in FMT, a PCA was conducted. In the PCA plots, PCA1 (31.4%, 42.4%, and 34.6%) showed higher contributions compared to PCA2 (19%, 19.5%, and 17.9%) in Pre-Monsoon, Monsoon, and Post-Monsoon, respectively. The water and sediment contributed positively to PCA1 during the Pre-Monsoon and Monsoon periods, but negatively during the Post-Monsoon period. This means That arsenic accumulation in most fish is significantly affected by the sediment and water in the Pre-Monsoon and monsoon periods. In Post-Monsoon, it depends on the sediment concentration. Additionally, the sampling points of the Upper-Part and Mid-Part contributed positively to PCA1 compared to the Lower-Part, indicating a significant effect of arsenic accumulation in the upper region compared to the lower region in the study area.

#### Impact of Morphometric distinctness, habitat distribution and tropic positions.

**Fig 5:**
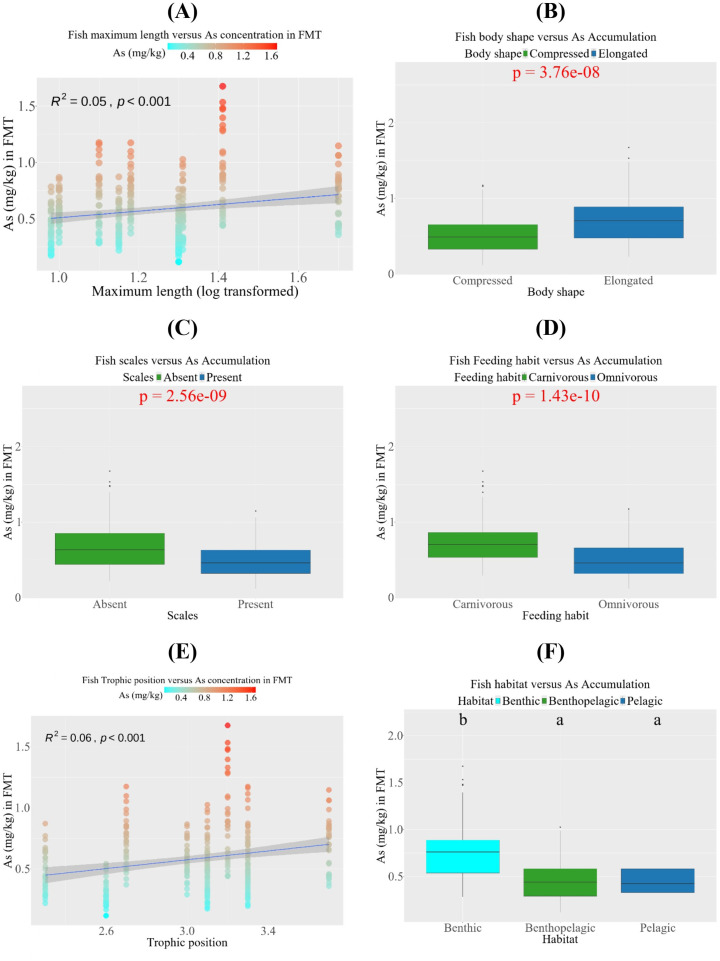
Impact of Morphometric distinctness, Habitat distribution and Tropic positions on Arsenic accumulation into FMT. Arsenic accumulation was calculated against different factors through maximum fish length (log-transformed) **(A)**, Body shape of the fish **(B)**, Presence of scales **(C)**, Fish feeding habit **(D)**, Fish trophic position **(E)**, Fish habitat **(F)**. The liner regarrisons **(A, E)**, Independent t-test **(B-D)**, and the ANOVA test with Tukey’s HSD **(F)** were done with a 99% confidence level, Where the p-value indicates the significance level at 0.001.

Fig 5 illustrates the influence of morphometric distinctness, trophic position, and habitat distribution on arsenic concentration in FMT. The linear regression between the maximum length of fish and arsenic accumulation in fish was insignificant (R² < 0.50, *p <* 0.001). So, the arsenic accumulation in SIS did not depend on the length at maturity. Subsequently, it rejected our hypothesis that, at maturity, the longer fish species might have more accumulation spaces than the shorter fish. Nonetheless, the result reinforces our assumption that elongated, scaleless fish tend to accumulate more arsenic than compressed, scaled fish. A significant effect (p < 0.001) of both factors on arsenic accumulation was observed based on the independent t-test. The higher arsenic concentrations in elongated-shaped fish may be attributed to their greater body surface area, which facilitates vertical uptake of arsenic from the surrounding water. Additionally, Fish scales help prevent arsenic from entering their muscle tissue. Our next attempt to investigate arsenic accumulation through feeding behaviors revealed significant differences between carnivores and omnivores (*p <* 0.001). Carnivores had higher arsenic concentrations than omnivorous fish, possibly due to the direct consumption of live foods, such as Zooplankton, whose body tissue stores arsenic [75]. Although the higher Trophic position of SIS fish shows a slightly higher concentration of arsenic, the relationship was insignificant (R^2^ < 0.50, *p <* 0.001) for consideration. Hence, the SIS fish species lie in a concise tropical position (between 2.3 and 3.7), so they may have had a non-significant linear relationship with arsenic accumulation. Finally, fish habitat distribution significantly differs among Pelagic, Benthopelagic, and Benthic Fish (*p <* 0.001), where Benthic fish lead in arsenic accumulation compared to the other fish species. The greater the depth of the fish habitat, the greater the arsenic accumulation in FMT. Monikh et al. argued that benthic fishes are directly connected to the bottom sediment and migrate less spatially than pelagic species, making it difficult for them to escape trace metals [76].

### Environmental and Ecological Risk Assessment

#### Pollution Level Assessment for Water and Sediment.

The potential risk assessment for water contamination by arsenic was evaluated using heavy metal pollution indices (HPI), contamination degree (CD), and the Nemerow pollution index (NPI), as depicted in Table 3. For HPI, the values ranged from 9.53 (L6) to 16.33 (C3), indicating low to moderate arsenic pollution. However, the low CD (CD < 6) and NPI (NPI < 1) values indicated low and insignificant pollution from arsenic. The combined indices indicated that the water in the Lower Meghna River was moderately polluted, depending on the sampling site. For sediment, all indices demonstrated values less than the minimum risk level (Igeo < 0, CF < 1, ERI < 110). The sediments indicated no pollution or lower pollution levels from arsenic. Therefore, drinking, using water for daily use, and taking aquatic animals as food might not risk human health from arsenic [14,30,67,69]. Moreover, aquatic organisms may not face potential risks due to their extended stay in the study area under the present status. [3,5,6]. However, the continuous presence of arsenic in water and sediments may pose a potential risk, as the levels are very close to the risk level.

**Table 3:** Potential Environmental and Ecological Risks of Arsenic through water and sediment in the Lower Meghna River.

Potential Environmental and Ecological Risks for Water						
Sampling sites	HPI	Pollution or risk level	CD	Pollution or risk level	NPI	Pollution or risk level
C1	15.80	Moderate pollution	0.16	Low pollution	0.05	Insignificant pollution
C2	10.40	Low pollution	0.10	Low pollution	0.02	Insignificant pollution
C3	16.33	Moderate pollution	0.16	Low pollution	0.08	Insignificant pollution
L4	14.07	Low pollution	0.14	Low pollution	0.06	Insignificant pollution
L5	12.47	Low pollution	0.12	Low pollution	0.03	Insignificant pollution
L6	9.53	Low pollution	0.10	Low pollution	0.03	Insignificant pollution
L7	12.93	Low pollution	0.13	Low pollution	0.03	Insignificant pollution
N8	11.27	Low pollution	0.11	Low pollution	0.02	Insignificant pollution
N9	9.80	Low pollution	0.10	Low pollution	0.02	Insignificant pollution
N10	15.27	Moderate pollution	0.15	Low pollution	0.04	Insignificant pollution
Potential Environmental and Ecological Risks for Sediment						
Sampling sites	Igeo	Pollution or risk level	CF	Pollution or risk level	ERI	Pollution or risk level
C1	−1.60	No pollution	0.49	Low pollution	4.95	Low Risk
C2	−1.76	No pollution	0.44	Low pollution	4.44	Low Risk
C3	−1.73	No pollution	0.45	Low pollution	4.53	Low Risk
L4	−1.58	No pollution	0.50	Low pollution	5.01	Low Risk
L5	−1.91	No pollution	0.40	Low pollution	3.98	Low Risk
L6	−1.59	No pollution	0.50	Low pollution	4.97	Low Risk
L7	−1.86	No pollution	0.41	Low pollution	4.14	Low Risk
N8	−1.67	No pollution	0.47	Low pollution	4.72	Low Risk
N9	−1.86	No pollution	0.41	Low pollution	4.14	Low Risk
N10	−2.03	No pollution	0.37	Low pollution	3.66	Low Risk

#### Metal bioaccumulation factor (MBAF).

Table 4 shows the metal bioaccumulation factors (MBAFs) in the SIS fish collected along the Lower Meghna River. Notably, Each fish species exhibited distinct bioaccumulative behaviors, reflecting interspecific differences in physiology, ecology, and exposure routes [3]. Based on MBAF_water_ values, all the fish demonstrated a lower probability of Bioaccumulation for arsenic (MBAF_water_ < 1000). Among the fish, the Ps (72.84) was the least, and the Ab (210.72) was the highest arsenic accumulative from water. On the other hand, fish were non-accumulative (MBAF_sediment _< 1) of arsenic from sediments. Moreover, MBAF_sediment_ values for arsenic followed a pattern like that observed in MBAF_water_, with Ps (0.05) exhibiting the lowest and Ab (0.16) the highest accumulation from sediments. The variation in trace metals like arsenic uptake among species is influenced by habitat preferences, feeding habits, body morphology, and metabolic strategies [4]. *Apocryptes bato*, a benthic, carnivorous, sluggish fish with an elongated body, exhibited the highest arsenic accumulation. In contrast, *Puntius sophore*, a fast-moving, scaled, omnivorous, benthopelagic fish with a compressed body shape, showed the lowest accumulation potential. These findings underscore the importance of ecological and biological traits in determining metal bioaccumulation patterns. It is also important to note that although MBAF_water_ values below 1000 are considered low based on Arnot & Gobas [41], such levels may still indicate significant accumulation depending on the duration of exposure and species-specific traits.

**Table 4:** Metal Bioaccumulation Factor (MBAF) through water and sediment in the Lower Meghna River.

Fish species	MBAF_Water_		MBAF_Sediment_	
	Mean value	accumulation range	Mean value	accumulation range
Mv	129.40	less probability	0.10	non-accumulative
Ps	72.84	less probability	0.05	non-accumulative
Gg	171.32	less probability	0.12	non-accumulative
Am	104.86	less probability	0.07	non-accumulative
Dd	153.03	less probability	0.10	non-accumulative
Lg	187.72	less probability	0.13	non-accumulative
Cc	97.37	less probability	0.07	non-accumulative
Gc	116.42	less probability	0.08	non-accumulative
Na	151.49	less probability	0.11	non-accumulative
Ab	210.72	less probability	0.16	non-accumulative

Mv, *Mystus vittatus;* Ps*, Puntius sophore;* Gg*, Glossogobius giuris;* Am*, Amblypharyngodon mola;* Dd*, Devario devario;* Lg*, Lepidocephalichthys guntea;* Cc*, Chela cachius;* Gc*, Gudusia chapra;* Na*, Neotropius atherinoides;* Ab*, Apocryptes bato.*

### Human health risk assessment

#### Estimation of daily intake (EDI).

The amount of arsenic in the river water, sediment, and Fish was used to calculate the harmful effects of exposure to humans via the three routes—ingestion, dermal contact, and inhalation. Table 5 displays the EDI value for the paths selected by the adult and child age groups. The table indicated that the children had comparatively greater EDI values than the adults for all the pathways and exposure media. Moreover, ingestion pathways showed more EDI values for water than dermal contact for adults and children. C3 and L6 had the highest and lowest EDI values for water, with mean values of 9.13E-09 (Ing) and 3.75E-10 (Der) for adults, and 4.26E-08 (Ing) and 8.59E-10 (Der) for children. The mean EDI values for sediment followed the pathways as ingestion (2.56E-06, 1.20E-05)> dermal contact (9.90E-07, 6.48E-06)>inhalation (4.26E-15, 1.99E-14) for adults and Children, respectively. There was no significant variation in EDI values in sampling stations for Fish through Ingestion; however, children demonstrated 4.5 times more EDI than adults. Regarding metal intake, the ingested path is the primary route to the EDI, supported by most similar studies [4,6,14,65]. Moreover, more arsenic was taken by children along the riverbank than by adults because youngsters have a higher exposure rate and, concerning their body weight, a larger consumption limit than adults [14].

**Table 5:** Estimated daily intake (mg/kg/d) of river water, Sediment and Fish for different exposure pathways for adults and children.

Stations	Water		Sediment			Fish
	EDI (Ing)	EDI (Der)	EDI (Ing)	EDI (Der)	EDI (Inh)	EDI (Ing)
Adults						
C1	1.13E-08	4.63E-10	2.85E-06	1.10E-06	4.73E-15	0.00052
C2	7.43E-09	3.05E-10	2.55E-06	9.86E-07	4.24E-15	0.00049
C3	1.17E-08	4.79E-10	2.61E-06	1.01E-06	4.33E-15	0.00049
L4	1.00E-08	4.12E-10	2.88E-06	1.11E-06	4.79E-15	0.00047
L5	8.90E-09	3.65E-10	2.29E-06	8.85E-07	3.81E-15	0.00044
L6	6.81E-09	2.79E-10	2.86E-06	1.10E-06	4.75E-15	0.00046
L7	9.24E-09	3.79E-10	2.38E-06	9.20E-07	3.96E-15	0.00043
N8	8.05E-09	3.30E-10	2.72E-06	1.05E-06	4.52E-15	0.00043
N9	7.00E-09	2.87E-10	2.38E-06	9.20E-07	3.96E-15	0.00044
N10	1.09E-08	4.48E-10	2.11E-06	8.14E-07	3.50E-15	0.00041
mean	9.13E-09	3.75E-10	2.56E-06	9.90E-07	4.26E-15	0.00046
Children						
C1	5.27E-08	1.06E-09	1.33E-05	7.20E-06	2.21E-14	0.00232
C2	3.47E-08	6.99E-10	1.19E-05	6.46E-06	1.98E-14	0.00218
C3	5.44E-08	1.10E-09	1.22E-05	6.59E-06	2.02E-14	0.00216
L4	4.69E-08	9.45E-10	1.35E-05	7.29E-06	2.24E-14	0.00208
L5	4.16E-08	8.38E-10	1.07E-05	5.80E-06	1.78E-14	0.00196
L6	3.18E-08	6.41E-10	1.33E-05	7.23E-06	2.22E-14	0.00204
L7	4.31E-08	8.69E-10	1.11E-05	6.03E-06	1.85E-14	0.00191
N8	3.76E-08	7.57E-10	1.27E-05	6.88E-06	2.11E-14	0.00192
N9	3.27E-08	6.59E-10	1.11E-05	6.03E-06	1.85E-14	0.00195
N10	5.09E-08	1.03E-09	9.83E-06	5.33E-06	1.63E-14	0.00182
mean	4.26E-08	8.59E-10	1.20E-05	6.48E-06	1.99E-14	0.00203

#### Non‑carcinogenic risk assessment (Target Hazard Quotient).

Table 6 shows the Target Hazard Quotient (THQ) for consuming water, sediment and targeted SIS fish species. A THQ value exceeding 1 suggests a potential non-carcinogenic health concern based on USEPA guidelines [54]. Arsenic exposure via water and sediment remained within safe limits (THQ < 1) for both adults and children. However, for fish consumption, the mean THQ exceeded 1 in both age groups, indicating a potential health risk. From water, the mean HQ for Ingestion and dermal contact is 3.04E-05 and 1.25E-06 for adults and 1.42E-04 and 2.86E-06 for Children. On the other hand, for sediment, the values were 8.54E-03, 3.30E-03, and 1.42E-11 for adults, and 3.99E-0, 2.16E-02, and 6.63E-11 for children through Ingestion, dermal contact, and inhalation, respectively. The mean THQ for Fish was four times higher for children than for adults, at 6.78 and 1.54, respectively, and the fish from the upper part demonstrated the highest THQ. The HI values followed a similar pattern to THQ, exceeding the safety threshold only in the fish consumption route. While THQ and HI provide valuable screening-level indicators of potential chronic health effects, they are not definitive predictors of actual risk. These indices are based on conservative assumptions and do not account for individual susceptibility or long-term cumulative effects; thus, they should be interpreted as precautionary indicators requiring further investigation rather than conclusive evidence of harm [4,14].

**Table 6:** Non-carcinogenic (THQ) and carcinogenic risk (CR) of metals for different age consumers of the river water, Sediment and Fish for different exposure pathways for adults and children.

Stations	Water				Sediment					Fish		
	HQ (Ing)	HQ (Der)	HI	CR	HQ (Ing)	HQ (Der)	HQ (Inh)	HI	CR	HQ (Ing)	HI	CR
Adults												
C1	3.76E-05	1.54E-06	3.92E-05	1.76E-08	9.49E-03	3.67E-03	1.58E-11	1.32E-02	5.92E-06	1.75	1.75	7.87E-04
C2	2.48E-05	1.02E-06	2.58E-05	1.16E-08	8.51E-03	3.29E-03	1.41E-11	1.18E-02	5.31E-06	1.65	1.65	7.42E-04
C3	3.89E-05	1.60E-06	4.05E-05	1.82E-08	8.68E-03	3.35E-03	1.44E-11	1.20E-02	5.42E-06	1.63	1.63	7.35E-04
L4	3.35E-05	1.37E-06	3.49E-05	1.57E-08	9.61E-03	3.71E-03	1.60E-11	1.33E-02	6.00E-06	1.57	1.57	7.06E-04
L5	2.97E-05	1.22E-06	3.09E-05	1.39E-08	7.64E-03	2.95E-03	1.27E-11	1.06E-02	4.77E-06	1.48	1.48	6.65E-04
L6	2.27E-05	9.32E-07	2.36E-05	1.06E-08	9.53E-03	3.68E-03	1.58E-11	1.32E-02	5.95E-06	1.54	1.54	6.94E-04
L7	3.08E-05	1.26E-06	3.21E-05	1.44E-08	7.94E-03	3.07E-03	1.32E-11	1.10E-02	4.95E-06	1.44	1.44	6.48E-04
N8	2.68E-05	1.10E-06	2.79E-05	1.26E-08	9.07E-03	3.50E-03	1.51E-11	1.26E-02	5.65E-06	1.45	1.45	6.52E-04
N9	2.33E-05	9.58E-07	2.43E-05	1.09E-08	7.94E-03	3.07E-03	1.32E-11	1.10E-02	4.95E-06	1.47	1.47	6.61E-04
N10	3.63E-05	1.49E-06	3.78E-05	1.70E-08	7.02E-03	2.71E-03	1.17E-11	9.74E-03	4.38E-06	1.38	1.38	6.19E-04
mean	3.04E-05	1.25E-06	3.17E-05	1.43E-08	8.54E-03	3.30E-03	1.42E-11	1.18E-02	5.33E-06	1.54	1.54	6.91E-04
Children												
C1	1.76E-04	3.54E-06	1.79E-04	8.06E-08	4.43E-02	2.40E-02	7.36E-11	6.83E-02	3.07E-05	7.72	7.72	3.47E-03
C2	1.16E-04	2.33E-06	1.18E-04	5.30E-08	3.97E-02	2.15E-02	6.60E-11	6.12E-02	2.76E-05	7.28	7.28	3.28E-03
C3	1.81E-04	3.66E-06	1.85E-04	8.33E-08	4.05E-02	2.20E-02	6.74E-11	6.25E-02	2.81E-05	7.21	7.21	3.24E-03
L4	1.56E-04	3.15E-06	1.59E-04	7.18E-08	4.49E-02	2.43E-02	7.46E-11	6.92E-02	3.11E-05	6.92	6.92	3.11E-03
L5	1.39E-04	2.79E-06	1.41E-04	6.36E-08	3.57E-02	1.93E-02	5.93E-11	5.50E-02	2.47E-05	6.52	6.52	2.93E-03
L6	1.06E-04	2.14E-06	1.08E-04	4.86E-08	4.45E-02	2.41E-02	7.39E-11	6.86E-02	3.09E-05	6.80	6.80	3.06E-03
L7	1.44E-04	2.90E-06	1.47E-04	6.60E-08	3.71E-02	2.01E-02	6.16E-11	5.72E-02	2.57E-05	6.36	6.36	2.86E-03
N8	1.25E-04	2.52E-06	1.28E-04	5.75E-08	4.23E-02	2.29E-02	7.03E-11	6.52E-02	2.94E-05	6.40	6.40	2.88E-03
N9	1.09E-04	2.20E-06	1.11E-04	5.00E-08	3.71E-02	2.01E-02	6.16E-11	5.72E-02	2.57E-05	6.49	6.49	2.92E-03
N10	1.70E-04	3.42E-06	1.73E-04	7.79E-08	3.28E-02	1.78E-02	5.45E-11	5.05E-02	2.27E-05	6.07	6.07	2.73E-03
mean	1.42E-04	2.86E-06	1.45E-04	6.52E-08	3.99E-02	2.16E-02	6.63E-11	6.15E-02	2.77E-05	6.78	6.78	3.05E-03

#### Carcinogenic risk assessment.

Significant variations in cancer risks (CRs) can occur depending on the metal type, exposure pathway, environmental media, and age group [32]. Our results indicate that children have higher CR levels than adults, consistent with their increased vulnerability to toxicants. Values below 10^−6^ indicate minimal CR exposure to the metals; in contrast, values above 10^−4^ indicate severe CR exposure. All the CR values, with means of 1.43E-08 and 6.52E-08 for adults and children, respectively, for water, were below 10^-6, indicating minimal CR exposure. For Sediment, the mean CR for adults and children were 5.33E-06 and 2.77E-05, which fell between 10^-6 and 10^-4, indicating a considerable carcinogenic risk. However, arsenic exposure via fish consumption presents a more serious concern. CR values for both adults and children significantly exceeded 10 ⁻ ⁴, with mean values of 6.91E-04 and 3.05E-03, respectively, indicating a substantial carcinogenic risk associated with dietary intake of fish from the study area. These findings align with previous studies reporting elevated cancer risks, particularly among children, from arsenic bioaccumulation in fish [3–6,65,75,77,78]. While this study did not perform probabilistic uncertainty analyses due to data limitations, the deterministic CR estimates highlight a pressing public health concern. Chronic arsenic exposure, especially through the consumption of contaminated fish, is linked to long-term adverse health outcomes including skin, lung, bladder, and kidney cancers, as well as developmental impairments in children [4]. Therefore, despite low arsenic levels in water and sediment, the bioaccumulated arsenic in Small Indigenous Species (SIS) fish poses a significant cancer risk to consumers, emphasizing the need for continuous monitoring, risk mitigation, and targeted public health interventions in the Lower Meghna River region.

### Implications for Public Health, Livelihoods, and Environmental Management

While quantitative analysis provides insight into arsenic distribution and health risks, it is equally important to consider the broader societal and ethical implications of these findings. The SIS fish species analysed in this study are not only ecologically significant but also culturally and nutritionally vital for riverside communities. In areas with high arsenic levels, eating these fish regularly can lead to long-term health problems like cancer, skin lesions, and developmental disorders, especially in children, pregnant women, and older people who are more likely to be susceptible. Beyond health risks, the reality or perception of contamination may reduce public confidence in wild-caught fish, leading to decreased market demand and economic hardship for small-scale fishers, processors, and vendors. This scenario raises grave ethical concerns, as affected populations may remain unaware of the risks due to limited access to scientific information or public health resources. As a result, arsenic pollution in riverine ecosystems needs to be fixed through integrating social responsibility with environmental policy. It is recommended that stricter regulations be enforced on industrial discharge and agricultural runoff, routine monitoring of food and water safety be implemented, and low-cost arsenic remediation technologies suitable for rural aquatic systems be developed. Public awareness campaigns and dietary advisories should also be implemented to ensure that local populations are informed and empowered to make safer food choices. These measures are essential not only to mitigate health risks but also to uphold environmental justice and protect the livelihoods of communities that depend on the Lower Meghna River. Employing an integrated management approach which incorporates science, policy, and public participation is crucial for protecting the environment and people’s health.

## Conclusion

This study assessed arsenic concentrations in water, sediment, and ten selected small indigenous species (SIS) of fish (FMTs) from the Lower Meghna River. While arsenic levels in water and sediment were generally within standard permissible limits, several fish species—Mystus vittatus, Glossogobius giuris, Lepidocephalichthys guntea, and Neotropius atherinoides—exceeded WHO guideline values for safe human consumption. Spatially, arsenic concentrations were highest in the upper reaches of the study area, though differences across zones were not statistically significant, potentially due to limited spatial resolution. Seasonally, the pre-monsoon period exhibited significantly elevated arsenic levels in all environmental compartments. Arsenic accumulation in fish tissues correlated positively with its concentration in water and sediment. Morphological traits—particularly body shape and the presence of scales—appeared to influence arsenic uptake more than size at maturity, though further studies with larger sample sizes are needed. Benthic, carnivorous species were found to accumulate more arsenic than omnivorous species from pelagic and benthopelagic zones. Pollution indices indicated low to moderate contamination in water and negligible pollution in sediment. Bioaccumulation assessments suggested limited transfer of arsenic from water to fish and minimal accumulation from sediment. However, health risk assessments revealed higher susceptibility in children due to their smaller size and physiological immaturity. While hazard quotients (HQ) and hazard indices (HI) for water and sediment remained below the safety threshold (HI < 1), fish consumption posed a non-carcinogenic risk—particularly for children. Carcinogenic risk (CR) estimates from water and sediment were within acceptable limits (10 ⁻ ⁶ to 10 ⁻ ⁴) but exceeded thresholds (>10 ⁻ ⁴) for fish consumption, indicating potential long-term cancer risk for high fish-consuming populations. Given these findings, it is strongly recommended that arsenic and other hazardous substances not be discharged into aquatic ecosystems without proper treatment. Regular monitoring and remediation strategies are critical to safeguarding ecological and public health in the Lower Meghna River region. Importantly, future studies should incorporate arsenic speciation, bioavailability assessments, and biological endpoints (e.g., biomarkers or bioindicators) to improve ecological risk evaluation. Additionally, integrating region-specific fish consumption data, public risk communication strategies, and stakeholder engagement will enhance the societal relevance and policy impact of future research.

### Limitations

This study has several limitations that should be considered when interpreting the results. First, arsenic speciation was not conducted; only total arsenic concentrations were measured in water, sediment, and fish tissue. Since inorganic arsenic, particularly in fish muscle, is significantly more toxic than organic forms, this may lead to over- or underestimation of health risks. Second, the deterministic approach used for health risk assessment does not incorporate variability or uncertainty in exposure parameters that probabilistic models like Monte Carlo simulation could better capture. Third, the Metal Bioaccumulation Factor (MBAF) thresholds applied were based on general literature values and may not fully reflect species- or site-specific dynamics. Fourth, while the study included 10 stations across three seasons, this sampling design may not fully represent the spatial and temporal variability of arsenic exposure in the Lower Meghna River system. Fifth, the health risk assessment focused solely on arsenic exposure through fish consumption, as arsenic concentrations in water and sediment were consistently below guideline values and unlikely to contribute significantly to chronic health effects. Consequently, cumulative exposure from all pathways, though potentially important in more contaminated areas, was not evaluated in this study. Sixth, the use of standard fish consumption rates did not account for specific dietary habits, portion sizes, or the exposure risk of sensitive subpopulations (e.g., children, pregnant women, elderly). These limitations stem largely from logistical and financial constraints. Future research should incorporate arsenic speciation, probabilistic modeling, expanded spatiotemporal sampling, site-specific bioaccumulation studies, and locally tailored fish consumption data. In high-risk regions, cumulative exposure assessments—including drinking water and sediment contact—are recommended to better inform public health policy.

## Supporting information

S1 Table Details of selected Small Indigenous Species (SIS) fish samples used in the study. (DOCX)

S2 Table Classification criteria with a value range for metal pollution indices. (DOCX)

S3 Table Explanation and values for the used Estimated daily intake (EDI) pathways. (DOCX)

S4 Table Minimal data set. (CSV)

S1 Glossary Glossary of Key Terms. (DOCX)

S1 Checklist Additional information regarding the ethical, cultural, and scientific considerations specific to inclusiveness in global research is included in the Supporting Information. (DOCX)
