# Supplementary material for: Arsenic bioaccumulation in fish of the lower meghna river: Seasonal dynamics, species sensitivity, and public health implications
Source: PLoS One. 2025 Sep 3;20(9):e0330602. doi: 10.1371/journal.pone.0330602 (PMC12407486; doi:10.1371/journal.pone.0330602)
Supplement: S1 Table — (DOCX) [file pone.0330602.s001.docx]

**S1 Table. Selected sample SIS fish with sample ID, their morphometric characteristics (Body shape, Presence of Scales, Maximum fish length), Food habits, and habitat types** **from Lower Meghna River, Bangladesh.**

| **Species** | **Common name** | **Sample ID** | **Body**  **shape** | **Scales** | **Max length (cm)** | **Migration**  **habit** | **Feeding**  **habit** | **Trophic level (point)** | **Habitat** |
| --- | --- | --- | --- | --- | --- | --- | --- | --- | --- |
| *Mystus vittatus* | Striped dwarf catfish | Mv | elongated | absent | 20.2 | Amphidromous | Omnivorous | 3.0-3.5 (3.1) | benthopelagic |
| *Puntius sophore* | Pool barb | Ps | compressed | present | 20 | Amphidromous | Omnivorous | 2.5-3.0 (2.6) | benthopelagic |
| *Glossogobius giuris* | Tank goby | Gg | elongated | present | 50 | Amphidromous | Omnivorous | 3.5-4 (3.7) | benthic |
| *Amblypharyngodon mola* | Mola carplet | Am | compressed | present | 20 | Potamodromous | Omnivorous | 3.0-3.5 (3.3) | pelagic |
| *Devario devario* | Sind Danio | Dd | compressed | present | 10 | Potamodromous | Carnivorous | 3.0-3.5 (3.0) | benthopelagic |
| *Lepidocephalichthys guntea* | Guntea loach | Lg | compressed | absent | 15 | Potamodromous | Carnivorous | 2.5-3.0 (2.7) | benthic |
| *Chela cachius* | silver hatchet chela | Cc | compressed | present | 9.6 | Amphidromous | Omnivorous | 3.0-3.5 (3.1) | benthopelagic |
| *Gudusia chapra* | Indian river shad | Gc | compressed | absent | 14 | Potamodromous | Omnivorous | 2.5-3.0 (2.3) | pelagic |
| *Neotropius atherinoides* | Indian potasi | Na | compressed | absent | 12.7 | Amphidromous | Omnivorous | 3.0-3.5 (3.3) | benthic |
| *Apocryptes bato* | Mudskipper | Ab | elongated | absent | 26 | Amphidromous | Carnivorous | 3.0-3.5 (3.2) | benthic |
