# Supplementary material for: Arsenic bioaccumulation in fish of the lower meghna river: Seasonal dynamics, species sensitivity, and public health implications
Source: PLoS One. 2025 Sep 3;20(9):e0330602. doi: 10.1371/journal.pone.0330602 (PMC12407486; doi:10.1371/journal.pone.0330602)
Supplement: S2 Table — (DOCX) [file pone.0330602.s002.docx]

**S2 Table. Classification criteria with a value range for metal pollution indices.**

| **Index** | **Pollution or risk level** | **Classification** | **Value** | **Reference** |
| --- | --- | --- | --- | --- |
| Heavy metal pollution index (HPI) | Low pollution | I | HPI<15 | [1–3] |
|  | Moderate pollution | II | 15 ≤HPI≤30 |  |
|  | Moderate to heavy pollution | III | 30 ≤HPI≤100 |  |
|  | Heavy pollution | IV | HPI<100 |  |
| Nemerow pollution index (NPI) | Insignificant pollution | I | NPI<1 | [1,2,4] |
|  | Slight pollution | II | 1 ≤NPI≤2.5 |  |
|  | Moderate pollution | III | 2.5 ≤NPI≤7 |  |
|  | Heavy pollution | IV | NPI>7 |  |
| Contamination degree (CD) | Low pollution | I | CD<6 | [1,2,5] |
|  | Moderate pollution | II | 6 ≤CD≤12 |  |
|  | Considerable pollution | III | 12 ≤CD≤24 |  |
|  | Very Heavy pollution | IV | CD>24 |  |
| Geo-accumulation index (Igeo) | No pollution | I | Igeo<0 | [6–8] |
|  | Negligible pollution | II | 0 ≤Igeo<1 |  |
|  | Endurable pollution | III | 1 ≤Igeo<2 |  |
|  | Endurable to strong pollution | IV | 2 ≤Igeo<3 |  |
|  | Strongly pollution | V | 3 ≤Igeo<4 |  |
|  | Strong to ultimate pollution | VI | 4 ≤Igeo<5 |  |
|  | Ultimate pollution | VII | 5 ≤Igeo |  |
| Contamination factor (CF) | Low pollution | I | CF<1 | [9–11] |
|  | Moderate pollution | II | 1 ≤CF≤3 |  |
|  | Considerable pollution | III | 3≤CF≤6 |  |
|  | Very Heavy pollution | IV | CF>6 |  |
| Ecological risk index (ERI) | Low Risk | I | ERI<110 | [1,2,5] |
|  | Moderate Risk | II | 110 ≤ERI≤200 |  |
|  | Considerable Risk | III | 200 ≤ERI≤400 |  |
|  | Very Heavy Risk | IV | ERI>400 |  |

References

1. Wen X, Lu J, Wu J, Lin Y, Luo Y. Influence of coastal groundwater salinization on the distribution and risks of heavy metals. Sci Total Environ. 2019;652: 267–277. doi:10.1016/j.scitotenv.2018.10.250

2. Tokatlı C, Varol M, Ustaoğlu F. Ecological and health risk assessment and quantitative source apportionment of dissolved metals in ponds used for drinking and irrigation purposes. Environ Sci Pollut Res. 2023;30: 52818–52829. doi:10.1007/s11356-023-26078-2

3. Zakir HM, Sharmin S, Akter A, Rahman MS. Assessment of health risk of heavy metals and water quality indices for irrigation and drinking suitability of waters: a case study of Jamalpur Sadar area, Bangladesh. Environ Adv. 2020;2. doi:10.1016/j.envadv.2020.100005

4. Vu CT, Lin C, Shern CC, Yeh G, Le VG, Tran HT. Contamination, ecological risk and source apportionment of heavy metals in sediments and water of a contaminated river in Taiwan. Ecol Indic. 2017;82: 32–42. doi:10.1016/j.ecolind.2017.06.008

5. Sharifi Z, Hossaini SMT, Renella G. Risk assessment for sediment and stream water polluted by heavy metals released by a municipal solid waste composting plant. J Geochemical Explor. 2016;169: 202–210. doi:10.1016/j.gexplo.2016.08.001

6. Zhuang Q, Li G, Liu Z. Distribution, source and pollution level of heavy metals in river sediments from South China. Catena. 2018;170: 386–396. doi:10.1016/j.catena.2018.06.037

7. Chen H, Teng Y, Lu S, Wang Y, Wang J. Contamination features and health risk of soil heavy metals in China. Sci Total Environ. 2015;512–513: 143–153. doi:10.1016/j.scitotenv.2015.01.025

8. Rahman MS, Hossain MB, Babu SMOF, Rahman M, Ahmed ASS, Jolly YN, et al. Source of metal contamination in sediment, their ecological risk, and phytoremediation ability of the studied mangrove plants in ship breaking area, Bangladesh. Mar Pollut Bull. 2019;141: 137–146. doi:10.1016/j.marpolbul.2019.02.032

9. Hakanson L. An ecological risk index for aquatic pollution control.a sedimentological approach. Water Res. 1980;14: 975–1001. doi:10.1016/0043-1354(80)90143-8

10. Loska K, Cebula J, Pelczar J, Wiechuła D, Kwapuliński J. Use of enrichment, and contamination factors together with geoaccumulation indexes to evaluate the content of Cd, Cu, and Ni in the Rybnik water reservoir in Poland. Water, Air, Soil Pollut. 1997;93: 347–365. doi:10.1007/bf02404766

11. Turekian KK, Wedepohl KH. Distribution of the elements in some major units of the earth’s crust. Bull Geol Soc Am. 1961;72: 175–192. doi:10.1130/0016-7606(1961)72[175:DOTEIS]2.0.CO;2
