# Supplementary material for: Arsenic bioaccumulation in fish of the lower meghna river: Seasonal dynamics, species sensitivity, and public health implications
Source: PLoS One. 2025 Sep 3;20(9):e0330602. doi: 10.1371/journal.pone.0330602 (PMC12407486; doi:10.1371/journal.pone.0330602)
Supplement: S1 Glossary — (DOCX) [file pone.0330602.s005.docx]

# Glossary of Key Terms

**SIS (Small Indigenous Species)**

A group of small-sized freshwater fish species commonly found and consumed in Bangladesh. They are culturally, nutritionally, and economically important.

**Arsenic (As)**

A toxic metalloid naturally occurring in the environment. In its inorganic form, it is more harmful to human health than in its organic form.

**Bioaccumulation Factor (BAF)**

The ratio of a metal's concentration in an organism to its concentration in the environment (water or sediment), used to assess bioaccumulation potential.

**MBAF (Metal Bioaccumulation Factor)**

A specific BAF that quantifies the accumulation of a particular metal (e.g., arsenic) in fish from water or sediment.

**FMT (Fish Muscle Tissue)**

The edible part of the fish (muscle) analyzed for metal contamination.

**THQ (Target Hazard Quotient)**

A non-carcinogenic risk indicator that estimates the potential health risk posed by long-term exposure to a contaminant. THQ > 1 suggests potential health concern.

**HI (Hazard Index)**

The sum of multiple THQ values across different exposure routes or contaminants. HI > 1 indicates a cumulative risk.

**CR (Carcinogenic Risk)**

A measure of the probability of an individual developing cancer over a lifetime due to exposure to a carcinogenic substance. A CR > 10⁻⁴ is considered a significant risk.

**ICP-MS (Inductively Coupled Plasma Mass Spectrometry)**

A highly sensitive analytical technique used to detect trace metals like arsenic in environmental samples.

**AAS (Atomic Absorption Spectroscopy)**

A laboratory technique used to measure concentrations of metals in samples.

**Speciation (Arsenic)**

The process of distinguishing between different chemical forms of arsenic (e.g., inorganic vs. organic), which vary in toxicity.

**Monte Carlo Simulation**

A statistical method used to model uncertainty by running multiple simulations with varying input parameters. Used in advanced risk assessment.

**Deterministic Risk Assessment**

A method that uses fixed input values (e.g., average ingestion rate) to calculate health risks, without considering variability.

**Probabilistic Risk Assessment**

A method that incorporates uncertainty and variability by using probability distributions for input parameters (e.g., body weight, exposure duration).

**Standard Reference Material (SRM)**

A certified material with known composition used to validate the accuracy of analytical procedures.
